# Trends in diabetes-related complications in Hong Kong, 2001–2016: a retrospective cohort study

**DOI:** 10.1186/s12933-020-01039-y

**Published:** 2020-05-12

**Authors:** Hongjiang Wu, Eric S. H. Lau, Aimin Yang, Ronald C. W. Ma, Alice P. S. Kong, Elaine Chow, Wing-Yee So, Juliana C. N. Chan, Andrea O. Y. Luk

**Affiliations:** 1 Department of Medicine and Therapeutics, The Chinese University of Hong Kong, Shatin, New Territories, Hong Kong Special Administrative Region People’s Republic of China; 2 Hong Kong Institute of Diabetes and Obesity, The Chinese University of Hong Kong, Shatin, New Territories, Hong Kong Special Administrative Region People’s Republic of China; 3 Li Ka Shing Institute of Health Sciences, The Chinese University of Hong Kong, Shatin, New Territories, Hong Kong Special Administrative Region People’s Republic of China; 4 Hong Kong Hospital Authority, Kowloon, Hong Kong Special Administrative Region People’s Republic of China

**Keywords:** Diabetes, Trends, Complications, Coronary heart disease, Stroke, Heart failure, Hyperglycaemic crisis, Lower-extremity amputation

## Abstract

**Background:**

Nationwide studies on contemporary trends in incidence of diabetes-related complications in Asia are lacking. We describe trends in incident coronary heart disease (CHD), stroke, heart failure, hyperglycaemic crisis, and lower-extremity amputation (LEA) in people with diabetes in Hong Kong between 2001 and 2016.

**Methods:**

The Hong Kong Diabetes Surveillance Database (HKDSD) is a territory-wide diabetes cohort identified from Hong Kong Hospital Authority electronic medical record system. We identified events of CHD, stroke, heart failure and hyperglycaemic crisis using hospital principal diagnosis codes at discharge and that of LEA using inpatient procedure codes. We used Joinpoint regression analysis to describe incidence trends by age and sex.

**Results:**

Between 2001 and 2016, a total of 390,071 men and 380,007 women aged 20 years or older with diabetes were included in the HKDSD. Event rates of CHD, stroke, heart failure, hyperglycaemic crisis and LEA declined by 69.4% (average annual percent change: − 7.6, 95% CI − 10.2, − 5.0), 70.3% (− 8.7, 95% CI − 9.8, − 7.5), 63.6% (− 6.4, 95% CI − 8.0, − 4.7), 59.1% (− 6.6, 95% CI − 12.4, − 0.44), and 67.5% (− 5.8, 95% CI − 7.2, − 4.4), in men and by 77.5% (− 9.9, 95% CI − 11.8, − 7.9), 74.5% (− 9.0, 95% CI − 9.6, − 8.4), 65.8% (− 7.0, 95% CI − 8.0, − 6.0), 81.7% (− 8.5, 95% CI − 10.5, − 6.5), and 72.7% (− 9.1. 95% CI − 12.2, − 5.8) in women, respectively, over a 16-year period in people with diabetes in Hong Kong. Joinpoint analysis identified greater declines in event rates of the five diabetes-related complications in the earlier one-third of study period and slowed down but remained significant until 2016. Event rates decreased for all age groups above 45 years for both sexes. There was no significant change in event rates in the group aged 20–44 years except for decline in hyperglycaemic crisis.

**Conclusions:**

The event rates of diabetes-related complications have declined substantially with no evidence of stabilization or increase in Hong Kong up to 2016. Improvements in outcome were observed for all age subgroups but not in young people with diabetes, calling for urgent action to improve quality of care to prevent complications in young people at risk.

## Background

The prevalence of diabetes is increasing worldwide, particularly in Asian countries which contribute over 60% of the global population with diabetes [1]. Uncontrolled diabetes dramatically increases the risk of macrovascular and microvascular complications including atherosclerotic cardiovascular disease, heart failure, lower extremity amputations (LEA), end-stage kidney disease and blindness, which are major contributors to disability and mortality in people with diabetes [2–4]. Complications from diabetes can be delayed or prevented by intensive regulation of blood glucose and other metabolic risk factors through lifestyle changes and pharmacological intervention [5, 6]. High income countries from Europe and North America reported large reduction in rates of most diabetes complications over a two-decade period since the 1990s although recent surveillance in the United States (U.S.) has identified a rebound in the incidence of LEA and hyperglycaemic crisis and stabilization of cardiovascular and kidney events in the last 5 years. There is a call for re-examination of trends in the incidence of diabetes-related complications given the changing characteristics of people with diabetes including progressively younger age of disease onset but longer living span and increasing rates of obesity especially in young people [7, 8]. On the other hand, recent introduction of sodium-glucose co-transporter-2 (SGLT-2) inhibitors and glucagon-like peptide-1 (GLP-1) receptor agonists which have been shown to prevent atherosclerotic cardiovascular diseases and hospitalisation for heart failure may affect the trends of some of these complications [9–11].

The establishment of the Hong Kong Hospital Authority (HA) which governs all public hospitals and the majority of government out-patient clinics in 1990 has positively transformed health service delivery in Hong Kong with improved integration of primary, secondary and tertiary care for 7.3 million people currently residing in Hong Kong. The Hong Kong Diabetes Surveillance Database (HKDSD) is a retrospective cohort of people with diabetes identified from the electronic medical record (EMR) system used in the Hong Kong HA and provides population-level statistics on health outcome among people with diabetes. Using the HKDSD, we previously reported a 15-year decline in absolute mortality in people with diabetes and relative mortality comparing people with and without diabetes [12]. Despite this encouraging trend, diabetes-related complications are major causes of morbidity, loss of societal productivity and poor quality of life. Yet, regionally representative trend data are scarce in Asia. In this study, we describe the trends in incidence rates of diabetes-related complications in Hong Kong Chinese with diabetes between 2001 and 2016.

## Methods

### Study population

The Hong Kong HA is a statutory administrative body formed in 1990, and manages all 43 public hospitals/institutions, 49 specialist clinics, and 73 general (primary care) out-patient clinics in Hong Kong. In 2000, the HA adopted a territory-wide EMR system to capture demographic information, diagnostic and procedure codes, laboratory results, prescription and consultation records of all people attending public hospitals and clinics through their unique Hong Kong Identity Card number [13]. This EMR system provides a standardized repository of all the clinical data and gives clinicians an integrated, longitudinal and lifelong view of people’s healthcare record. In the period 2015 to 2016, the HA EMR system recorded about 6.2 million attendees in general out-patient clinics, 7.2 million attendees in specialist clinics and 1.1 million inpatient services, covering ~ 90% of total medical services in Hong Kong [14]. The HKDSD is a population-based cohort of people with diabetes identified from the HA EMR system. Diabetes was ascertained based on one or more of the following qualifying criteria: (1) recording of diagnostic code of diabetes based on International Classification of Diseases, Ninth Revision (ICD-9) code of 250.xx; (2) recording of diagnostic code of diabetes according to the Revised Edition of the International Classification of Primary Care, World Organization of National Colleges, Academies, and Academic Associations of General Practitioners/Family Physicians code T89 or code T90; (3) glycated haemoglobin (HbA1c) ≥ 6.5% (48 mmol/mol) in any one available measurement; (4) fasting plasma glucose ≥ 7.0 mmol/L in any one available measurement; (5) ever prescribed glucose-lowering drugs; or (6) long-term prescription of insulin (≥ 28 days). Women with gestational diabetes were not included in the HKDSD based on the diagnoses occurring 9 months before or 6 months after delivery (ICD-9 code 72-75) or within 9 months of any pregnancy-related encounter (ICD-9 codes 630-676). However, women with subsequent episodes that met the criteria of diabetes occurring outside the context of any obstetric events would be included. This study was approved by the local clinical research ethics committee.

### Outcome ascertainment

We included five diabetes-related complications in our study, including coronary heart disease (CHD), stroke, heart failure, hyperglycaemic crisis (including diabetic ketoacidosis and hyperglycaemic hyperosmolar state), and LEA, which are major disease burdens. We identified events of CHD (ICD-9 codes: 410-414), stroke (ICD-9 codes: 430-434, 436-438), heart failure (ICD-9 codes: 428), and hyperglycaemic crisis (ICD-9 codes: 250.1 and 250.2) using hospital principal diagnosis codes at discharge. We identified LEA events using inpatient procedure codes (ICD-9 procedure codes: 84.10-84.19, excluding discharges with a traumatic amputation diagnosis code: 895-897). Minor LEA (ICD-9 procedure codes: 84.11-84.12) was defined by LEA below the ankle and major LEA (ICD-9 procedure codes: 84.13-84.19) was defined by LEA at the ankle or above. We excluded day-case admissions defined as people admitted and discharged on the same day in our analyses, as these day-case admissions are mainly for assessment, review and/or non-invasive intervention (e.g. intravenous infusion for medications).

### Statistical analysis

We limited our analyses to 2001 to 2016 because of increasing completeness of diabetes records in the HKDSD since 2001. We included adults aged 20 years and older, as few people had diabetes and developed complications who were younger than 20 years of age. We performed all analyses separately for men and women, and for age-subgroups: 20–44, 45–64, 65–74 and ≥ 75 years, to describe the effects of age and sex on the trend estimates. We calculated age-standardized annual incidence rate of diabetes-related complications and used the 2016 Hong Kong Census mid-year population as the standard. We counted the total number of events as the numerator and used the mid-year population estimates in the HKDSD as the denominator. Incidence rates were expressed as number of events per 10,000 people with diabetes. We used Joinpoint regression analysis to describe the trends in age-standardized incidence rates of diabetes-related complications, and to investigate whether there were time points at which significant changes in trends occurred. We allowed a maximum of two joinpoints in the models based on the number of observations (16 calendar years) and used a Monte Carlo permutation method for model selection [15]. We set the minimum number of observations from a joinpoint to either end of the data as two, and the minimum number of observations between two joinpoints as three to reduce over-fitting of the models. We calculated the average annual percent change (AAPC) for the full study period (2001 to 2016) and the annual percent change (APC) for each linear trend segment detected from the Joinpoint regression models. We have previously reported changes in risk factor control over time in the HKDSD [12]. Here, we additionally calculated annual age-standardized prevalence of statin use in people with diabetes, as statins are effective treatment in primary and secondary prevention of cardiovascular diseases [16, 17]. Additionally, we calculated annual age-standardized prevalence of use of new glucose-lowering drugs including dipeptidyl-peptidase-4 (DPP-4) inhibitors, GLP-1 receptor agonists and SGLT2 inhibitors [18, 19].

We performed two sensitivity analyses. First, we repeated the analysis by excluding people newly included in the HKDSD in each study year from the denominator, to assess the effect of a progressive increase in diabetes detection over time through improved diagnostics and screening efforts on trends in rates of diabetes-related complications. Second, we repeated the analysis excluding 2426 people with type 1 diabetes of onset date between 2002 and 2015, identified using a validated algorithm [20]. Due to restriction of the algorithm, we could not identify people with prevalent type 1 diabetes of onset date before 2002. We performed all analyses using R (version 3.5.3, Vienna, Austria) or Joinpoint Regression Program (version 4.7.0.0; Statistical Methodology and Applications Branch, Surveillance Research Program, National Cancer Institute). A two-tailed *P* value less than 0.05 was considered statistically significant.

## Results

This study included 390,071 men and 380,007 women with diabetes aged 20 years or older in Hong Kong between 2001 and 2016. The number of men and women living with diabetes in the middle of the year increased by approximately 4.5 and 3.5 times compared to 2001 during this 16-year period, respectively (Additional file 1: Figure S1 and Table S1). Between 2001 and 2016, there was an increase in mean age but a decrease in fasting glucose, HbA1c and low-density lipoprotein (LDL)-cholesterol level among people in the HKDSD (Additional file 1: Table S1). A total of 146,187 CHD events, 127,567 stroke events, 121,499 heart failure events, 5230 hyperglycaemic crisis events and 10,294 LEA events were recorded in the HKDSD. The total event number of the five diabetes-related complications also rose from 2001 to 2016 albeit to a lesser extent, with the greatest relative increase in heart failure in both sexes (Additional file 1: Figure S2).

**Fig. 1:**
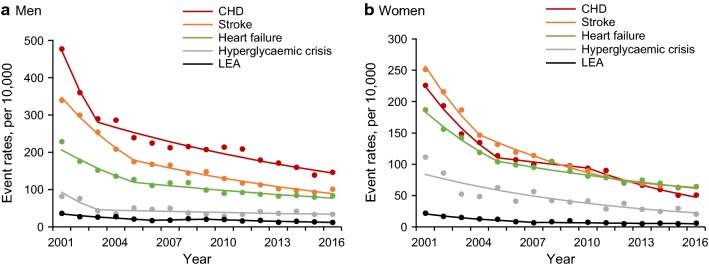
Age-standardized event rates of diabetes-related complications in men and women with diabetes. Dots are observed event rates. Solid lines are modeled event rates from the Joinpoint regression analysis

Men consistently had higher event rates of CHD, stroke, heart failure and LEA than women across all age groups although the difference was less prominent in people aged ≥ 75 years (Fig. 1, Additional file 1: Tables S2 and S3). The event rates of hyperglycaemic crisis were higher in women than men in the earlier part of the study period and reversed after 2011. The event rates of diabetes-related complications increased with increasing age group, with the exception of hyperglycaemic crisis, which was greatest in the youngest age group of 20–44 years in both sexes (Additional file 1: Tables S2 and S3).

### Trends in event rates by sex

From 2001 to 2016, the event rates declined significantly by 69.4% for CHD, 70.3% for stroke, 63.6% for heart failure, 59.1% for hyperglycaemic crisis and 67.5% for LEA in men (Fig. 1 and Table 1). The corresponding declines in women was 77.5% for CHD, 74.5% for stroke, 65.8% for heart failure, 81.7% for hyperglycaemic crisis and 72.7% for LEA (Fig. 1 and Table 2). The point estimates of all the AAPCs for declines in diabetes-related complications were higher in women than men, but generally with overlapping 95% CIs. The greatest AAPC was observed for stroke (− 8.7, 95% CI − 9.8, − 7.5) followed by CHD (− 7.6, 95% CI − 10.2, − 5.0) in men, and for CHD (− 9.9, 95% CI − 11.8, − 7.9) followed by LEA (− 9.1, 95% CI − 12.2, − 5.8) in women. The smallest AAPC was observed for LEA in men (− 5.8, 95% CI − 7.2, − 4.4) and for heart failure in women (− 7.0, 95% CI − 8.0, − 6.0). When stratified by anatomical level, greater declines were observed for minor LEA than major LEA in both sexes (Additional file 1: Table S4). Joinpoint regression analysis showed that the declines in event rates of the five diabetes-related complications were generally more marked in the first 3 to 7 years of the surveillance period and slowed down but were still significant until 2016 for both sexes (Fig. 1, Tables 1 and 2). Sensitivity analysis excluding people newly included in the HKDSD in each year (Additional file 1: Figure S3) or people with incident type 1 diabetes (data are not shown) yielded similar declining trends in rates of diabetes-related complications.

**Table 1:** Joinpoint analysis of trends in age-standardized event rates of diabetes-related complications in men with diabetes

Complications	Event rates (per 10,000)			Time period 1		Time period 2		Time period 3	
	2001	2016	AAPC (95% CI)	Year	APC (95% CI)	Year	APC (95% CI)	Year	APC (95% CI)
CHD									
All ages	477.3	146.0	− 7.6 (− 10.2, − 5.0)*	2001–2003	− 23.1 (− 38.8, − 3.3)*	2003–2016	− 5.0 (− 6.0, − 4.0)*		
20–44 years	155.9	55.1	− 7.3 (− 15.0, 1.1)	2001–2003	− 32.2 (− 66.1, 35.7)	2003–2016	− 2.7 (− 6.0, 0.68)		
45–64 years	651.9	199.8	− 7.6 (− 9.5, − 5.7)*	2001–2003	− 22.6 (− 32.2, − 11.7)*	2003–2007	− 7.5 (− 12.9, − 1.9)*	2007–2016	− 3.9 (− 4.9, − 3.0)*
65–74 years	914.3	217.1	− 9.0 (− 11.1, − 6.8)*	2001–2006	− 13.7 (− 15.8, − 11.5)*	2006–2010	− 3.1 (− 8.4, 2.6)	2010–2016	− 8.9 (− 10.5, − 7.3)*
≥ 75 years	813.3	281.1	− 6.5 (− 8.7, − 4.2)*	2001–2006	− 9.3 (− 13.7, − 4.6)*	2006–2010	3.6 (− 4.5, 12.4)	2010–2016	− 10.4 (− 12.5, − 8.3)*
Stroke									
All ages	339.5	100.9	− 8.7 (− 9.8, − 7.5)*	2001–2005	− 15.3 (− 19.0, − 11.3)*	2005–2016	− 6.2 (− 7.1, − 5.2)*		
20–44 years	56.2	50.3	− 1.8 (–4.6, 1.1)	2001–2016	− 1.8 (− 4.6, 1.1)				
45–64 years	338.2	99.7	− 8.3 (− 9.6, − 7.1)*	2001–2005	− 16.3 (− 20.4, − 12.0)*	2005–2016	− 5.3 (− 6.1, − 4.4)*		
65–74 years	788.5	157.5	− 10.5 (− 11.5, − 9.5)*	2001–2005	− 15.4 (− 18.8, − 12.0)*	2005–2016	− 8.7 (− 9.5, − 7.9)*		
≥ 75 years	1224.8	289.0	− 9.6 (− 10.6, − 8.6)*	2001–2005	− 14.8 (− 18.3, − 11.1)*	2005–2016	− 7.6 (− 8.3, − 7.0)*		
Heart failure									
All ages	228.6	83.1	− 6.4 (− 8.0, − 4.7)*	2001–2005	− 12.8 (− 18.3, − 7.0)*	2005–2016	− 3.9 (− 5.2, − 2.7)*		
20–44 years	41.6	30.8	1.2 (− 2.4, 4.8)	2001–2016	1.2 (− 2.4, 4.8)				
45–64 years	147.5	56.6	− 5.8 (− 8.1, − 3.5)*	2001–2006	− 13.7 (− 19.7, − 7.3)*	2006–2016	− 1.6 (− 3.7, 0.52)		
65–74 years	431.9	116.4	− 8.5 (− 10.0, − 7.1)*	2001–2009	− 11.3 (− 13.3, − 9.2)*	2009–2016	− 5.3 (− 7.9, − 2.7)*		
≥ 75 years	1259.2	413.4	− 6.9 (− 8.7, − 5.1)*	2001–2005	− 13.0 (− 19.3, − 6.2)*				
Hyperglycaemic crisis									
All ages	81.5	33.3	− 6.6 (− 12.4, − 0.44)*	2001–2003	− 30.0 (− 57.9, 16.6)	2003–2016	− 2.4 (− 4.9, 0.24)		
20–44 years	151.7	66.0	− 3.9 (− 6.2, − 1.5)*	2001–2016	− 3.9 (− 6.2, − 1.5)*				
45–64 years	25.4	10.1	− 7.0 (− 10.6, − 3.2)*	2001–2005	− 22.3 (− 33.0, − 9.9)*	2005–2016	− 0.7 (− 3.5, 2.2)		
65–74 years	23.2	4.0	− 12.2 (− 16.3, − 7.9)*	2001–2003	− 42.7 (− 60.4, − 17.2)*	2003–2016	− 6.2 (− 8.4, − 4.0)*		
≥ 75 years	42.3	5.5	− 13.2 (− 17.8, − 8.5)*	2001–2004	− 31.4 (− 48.0, − 9.5)*	2004–2016	− 8.0 (− 10.7, − 5.3)*		
LEA									
All ages	36.0	11.7	− 5.8 (− 7.2, − 4.4)*	2001–2016	− 5.8 (− 7.2, − 4.4)*				
20–44 years	14.1	3.8	− 7.4 (− 19.5, 6.5)	2001–2006	− 8.7 (− 22.4, 7.5)	2006–2010	14.5 (− 17.5, 59.0)	2010–2016	− 18.9 (− 27.5, − 9.3)*
45–64 years	37.5	18.4	− 2.8 (− 4.7, − 0.91)*	2001–2016	− 2.8 (− 4.7, − 0.91)*				
65–74 years	48.0	16.4	− 8.0 (− 9.5, − 6.5)*	2001–2016	− 8.0 (− 9.5, − 6.5)*				
≥ 75 years	123.0	16.6	− 10.5 (− 12.1, − 8.9)*	2001–2016	− 10.5 (− 12.1, − 8.9)*				

*CHD* coronary heart disease, *LEA* lower-extremity amputation, *AAPC* average annual percent change, *APC* annual percent change

* *P* < 0.05

**Table 2:** Joinpoint analysis of trends in age-standardized event rates of diabetes-related complications in women with diabetes

Complications	Event rates (per 10,000)			Time period 1		Time period 2		Time period 3	
	2001	2016	AAPC (95% CI)	Year	APC (95% CI)	Year	APC (95% CI)	Year	APC (95% CI)
CHD									
All ages	225.8	50.8	− 9.9 (− 11.8, − 7.9)*	2001–2005	− 16.3 (− 20.0, − 12.4)*	2005–2010	− 3.2 (− 8.4, 2.4)	2010–2016	− 10.8 (− 13.6, − 7.9)*
20–44 years	18.6	12.5	− 2.8 (− 7.6, 2.3)	2001–2016	− 2.8 (− 7.6, 2.3)				
45–64 years	240.7	43.0	− 10.9 (− 12.1, − 9.8)*	2001–2005	− 19.6 (− 23.2, − 15.8)*	2005–2016	− 7.6 (− 8.6, − 6.6)*		
65–74 years	623.5	94.0	− 11.9 (− 12.8, − 10.9)*	2001–2005	− 18.5 (− 20.4, − 16.5)*	2005–2011	− 6.0 (− 7.9, − 4.3)*	2011–2016	− 13.0 (− 14.8, − 11.2)*
≥ 75 years	727.3	223.2	− 7.7 (− 8.7, − 6.6)*	2001–2005	− 10.1 (− 13.0, − 7.1)*	2005–2010	− 0.83 (− 3.2, 1.6)	2010–2016	− 11.4 (− 12.5, − 10.3)*
Stroke									
All ages	251.3	64.2	− 9.0 (− 9.6, − 8.4)*	2001–2004	− 16.9 (− 19.0, − 14.8)*	2004–2012	− 8.5 (− 9.1, − 7.9)*	2012–2016	− 3.7 (− 5.4, − 2.0)*
20–44 years	41.5	28.2	− 4.4 (− 7.5, − 1.1)*	2001–2016	− 4.4 (− 7.5, − 1.1)*				
45–64 years	216.1	52.3	− 9.2 (− 10.5, − 8.0)*	2001–2006	− 16.2 (− 19.3, − 12.9)*	2006–2016	− 5.5 (− 6.8, − 4.2)*		
65–74 years	590.2	100.3	− 11.5 (− 13.0, − 10.0)*	2001–2004	− 19.2 (− 25.7, − 12.2)*	2004–2016	− 9.5 (− 10.5, − 8.5)*		
≥ 75 years	1044.8	249.7	− 9.4 (− 10.7, − 8.0)*	2001–2005	− 13.5 (− 18.3, − 8.4)*	2005–2016	− 7.8 (− 8.7, − 6.9)*		
Heart failure									
All ages	187.0	63.9	− 7.0 (− 8.0, − 6.0)*	2001–2005	− 13.0 (− 16.1, − 9.8)*	2005–2016	− 4.7 (− 5.7, − 3.8)*		
20–44 years	9.2	13.9	3.4 (− 2.4, 9.4)	2001–2016	3.4 (− 2.4, 9.4)				
45–64 years	87.4	25.6	− 8.2 (− 10.0, − 6.2)*	2001–2006	− 15.3 (− 20.0, − 10.3)*	2006–2016	− 4.4 (− 6.3, − 2.5)*		
65–74 years	371.0	79.1	− 9.8 (− 11.2, − 8.4)*	2001–2005	− 15.7 (− 20.3, − 10.9)*	2005–2016	− 7.6 (− 8.8, − 6.4)*		
≥ 75 years	1273.3	454.3	− 5.9 (− 6.8, − 5.1)*	2001–2016	− 5.9 (− 6.8, − 5.1)*				
Hyperglycaemic crisis									
All ages	111.3	20.4	− 8.5 (− 10.5, − 6.5)*	2001–2016	− 8.5 (− 10.5, − 6.5)*				
20–44 years	225.1	36.0	− 8.7 (− 10.8, − 6.6)*	2001–2016	− 8.7 (− 10.8, − 6.6)*				
45–64 years	22.6	10.0	− 5.9 (− 9.1, − 2.6)*	2001–2008	− 12.4 (− 17.8, − 6.7)*	2008–2016	0.22 (− 4.5, 5.1)		
65–74 years	8.5	4.0	− 7.5 (− 10.5, − 4.4)*	2001–2016	− 7.5 (− 10.5, − 4.4)*				
≥ 75 years	48.7	6.2	− 11.2 (− 15.2, − 6.9)*	2001–2006	− 28.2 (− 36.8, − 18.4)*	2006–2016	− 1.3 (− 5.7, 3.4)		
LEA									
All ages	21.6	5.9	− 9.1 (− 12.2, − 5.8)*	2001–2007	− 16.2 (− 22.1, − 9.9)*	2007–2016	− 4.0 (− 8.1, 0.32)		
20–44 years	9.4	4.9	Not available	2001− 2006	− 15.5 (− 32.5, 5.9)	2008–2016	− 0.37 (− 16.5, 18.9)		
45–64 years	11.9	5.2	− 6.4 (− 10.0, − 2.7)*	2001–2006	− 15.0 (− 23.7, − 5.2)*	2006–2016	− 1.8 (− 5.5, 2.0)		
65–74 years	39.2	5.1	− 11.8 (− 14.2, − 9.4)*	2001–2016	− 11.8 (− 14.2, − 9.4)*				
≥ 75 years	102.2	14.4	− 11.5 (− 12.7, − 10.2)*	2001–2016	− 11.5 (− 12.7, − 10.2)*				

*CHD* coronary heart disease, *LEA* lower-extremity amputation, *AAPC* average annual percent change, *APC* annual percent change

* *P* < 0.05. As no women aged 20–44 years had amputation event in 2007, trend analysis was performed for 2001–2006 and 2008–2016, separately

### Trends in event rates by age

**Fig. 2:**
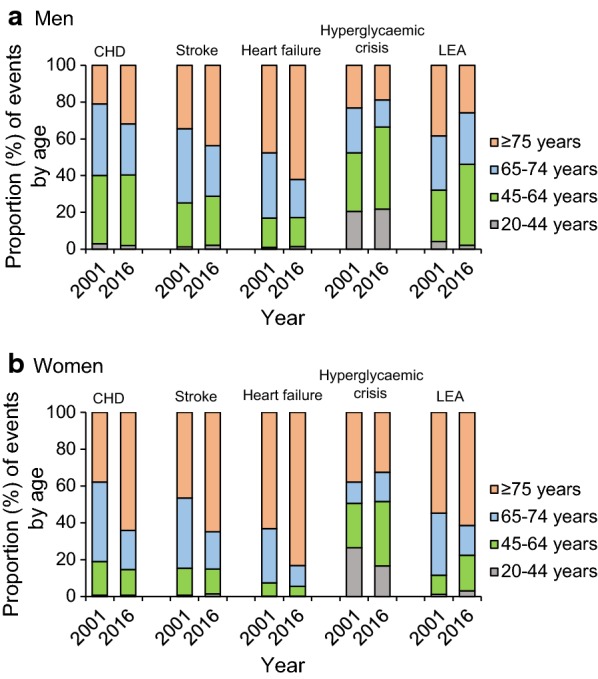
Proportion of events of diabetes-related complications by age in men and women with diabetes

The declining trends in event rates of diabetes-related complications varied by age group. In young men and women aged 20–44 years, event rates for most diabetes-related complications were stable except for a decline in hyperglycaemic crisis in both men (AAPC: − 3.9, 95% CI − 6.2, − 1.5) and women (AAPC: − 8.7, 95% CI − 10.8, − 6.6), and a decline in stroke (AAPC: − 4.4, 95% CI − 7.5, − 1.1) in women (Table 1 and 2). In other age-subgroups, the pattern of decreasing rates of the five diabetes-related complications was generally similar to that in the whole age group. The largest AAPCs for CHD, stroke and heart failure were observed in the age group of 65–74 year in both sexes. The largest AAPCs for LEA were in the age group of ≥ 75 years in men and 65–74 years in women, and for hyperglycaemic crisis, in the age group of ≥ 75 years in both sexes (Tables 1 and 2). The differential declines across age groups resulted in a decrease in proportion of people aged 65–74 years accompanied by a marked increase in proportion of people aged ≥ 75 years contributing to CHD, stroke and heart failure events between 2001 and 2016 (Fig. 2). In 2001, men aged ≥ 75 years accounted for 21.0% of CHD, 34.4% of stroke and 47.6% of heart failure events, and the proportions increased to 31.8%, 43.7% and 61.9% respectively in 2016. For women, the contribution of the oldest age group to CHD, stroke and heart failure events increased from 37.8%, 46.6% and 63.2% in 2001, to 64.1%, 64.9% and 83.3% in 2016, respectively. For LEA, an increase in proportion of people aged 45–64 years was observed. There was no clear changing pattern in relation to age for hyperglycaemic crisis. The youngest age group of 20–44 years contributed to a larger proportion of hyperglycaemic crisis events compared with contribution to other complications. The details of trends in proportion of the five diabetes-related complications by age group from 2001 to 2016 are shown in Additional file 1: Figures S4–S8.

### Trends in prevalence of statin and use of new glucose-lowering drugs

In 2001, 11.0% of men and 10.4% of women with diabetes ever used statins (Additional file 1: Table S5). The prevalence of statin use was constant before 2008 and increased dramatically thereafter to 44.5% in men and 40.2% in women in 2016. The increasing pattern was similar across all age groups but with a much lower prevalence in young people aged 20–44 years, which was 27.6% in men and 18.5% in women in 2016. New classes of glucose-lowering drugs including DPP-4 inhibitors, GLP-1 receptor agonists and SGLT2 inhibitors were available in Hong Kong from 2007, 2011 and 2015, respectively. The prevalence of DPP-4 inhibitors use has increased to 7.8% in men and 6.4% in women in 2016 (Additional file 1: Table S6). However, the prevalence of GLP-1 receptor agonists and SGLT2 inhibitors use remained very low. In 2016, less than 0.2% of people with diabetes were prescribed GLP-1 receptor agonists, and about 0.8% of people were prescribed SGLT2 inhibitors.

## Discussion

In this retrospective analysis of territory-wide data of Hong Kong Chinese aged 20 years and older with diabetes, we revealed substantial declines in event rates of five major diabetes-related complications including CHD, stroke, heart failure, hyperglycaemic crisis and LEA from 2001 to 2016. The declines were greater in the earlier part of the surveillance and diminished thereafter but no plateauing or increase in event rates was detected. The decrease in event rates of most complications were restricted to people aged 45 years and older. The younger group aged 20–44 years experienced decrease in rates of hyperglycaemic crisis but not in other complications. Our findings provide important insights into the epidemiology of diabetes-related complications in Asia and indicate improvements in the quality of care of most people with diabetes in Hong Kong in recent years [21].

### Declining trends in diabetes-related complications

Declines in rates of complications among people with diabetes have been reported in many western countries including the United Kingdom, Sweden and Canada up until the mid- to early 2000s [22]. Most recently, however, national statistics in the U.S. revealed increasing trends for several diabetes-related clinical events including acute myocardial infarction, stroke, LEA and hyperglycaemic crisis between 2010 and 2015, and the increase was the most apparent in the young and middle-aged people [23]. Comparable population-level statistics from Asian countries are limited [24–26]. A study in Taiwan showed an overall decline in hospitalization for diabetic ketoacidosis from 6.0 per 1000 person-years in 1997 to 5.0 in 2005 but with non-significant changes in young men and even rising trends in young women aged ˂35 years [24]. In Korea, hospital admission rates fell by 29.5% for CHD and 37.3% for acute myocardial infarction among people with type 2 diabetes between 2006 and 2013 based on the National Health Insurance database [25].

Causes for the declines in event rates of diabetes-related complications in Hong Kong are likely to be multifactorial, including changes in clinical care, healthcare delivery system, socioeconomic environment, and individuals’ health-related behaviours. Between 1990s and the early millennium, in response to the rapidly growing burden of diabetes in Asia, a series of quality improvement programs were introduced in Hong Kong [21]. These include establishment of nurse-led diabetes centres in major public hospitals in 2000 to provide regular structured assessment of risk factors and complications to people with diabetes [27]. In 2007, the program was extended to all public primary care clinics as the diabetes Risk Assessment and Management Program (RAMP) to support clinical decision making and to promote patient adherence to self-care and medication [21, 28, 29]. These territory-wide changes in care organization along with advances in pharmacotherapy have resulted in better control of metabolic risk factors over the last two decades [27]. In the present study, we further detected a marked increase in the usage of statins which is likely to contribute to the improved outcomes during this time period given the proven cardiovascular benefits of statins [30].

Other contributing factors include health promotion campaigns and complementary policy changes aimed at reducing behavioural risk factors as evidenced by a stable trend in the prevalence of overweight or obesity and a decreased prevalence of cigarette smoking and physical inactivity in Hong Kong [31, 32]. Positive changes in socioeconomic status of people with diabetes could also affect health outcomes through improvement in health literacy, disease awareness and lifestyles [33–35]. These societal and personal factors may bring about greater awareness leading to earlier detection and more timely intervention although we were unable to quantify the impacts of these factors on the trend estimates.

### More marked declines in earlier part of the study period

The faster declines in rates of diabetes-related complications in the earlier than latter part of the study period concord with the more marked decrease in all-cause mortality among people with diabetes in the period between 2001 and 2006 than between 2006 and 2016 in Hong Kong [12]. The reason for the slowing down of improvement is not clear. One possible explanation could be that improved survival has resulted in increasing number of people with long diabetes duration and living to older ages who are at inherently higher risks of developing complications [36]. Long disease duration is a major determinant for diabetes-related complications although we do not have this data for analysis in this administrative database [27]. It is also conceivable that healthcare reforms beginning in the 1990s yielded the greatest benefits during the period around its inception which then lessen over time.

### Unchanged rates of vascular complications in young people with diabetes

In clinic-based population, one in five adults with type 2 diabetes in Asia are diagnosed before the age of 40 years [37]. Contrary to a stabilizing trend of incidence of diabetes in the older age group, incidence of young-onset diabetes has continued to increase in Hong Kong. In a multinational study in Asia, people with young-onset diabetes had more adverse metabolic profiles and were less likely to achieve treatment goals and to receive organ-protective drugs such as statins and renin-angiotensin system inhibitors than those with late-onset diabetes [37]. In the present study, 18% of women and 27% of men in the 20–44 age group were prescribed statins compared to over 50% in other age groups. In our previous analysis on mortality rate during the same period, improvements in HbA1c and LDL-cholesterol levels in young people were smaller than their older counterparts [12]. This was reflected by the lack of reduction in all-cause mortality in young people who had fivefold higher risk of mortality compared with age-matched people without diabetes. In a separate study, we estimated that people with young-onset diabetes spent an average of 97 days in hospital up to the age of 75 years, which are substantially reduced by 30% if all risk factors are optimized and by 70% if the onset of diabetes can be delayed to after the age of 65 years [38].

### Changes in contribution of different age groups to diabetes-related complications

We observed a significant shift in contribution to most cardiovascular diseases including coronary heart disease, stroke and heart failure from people aged 65–74 years to the oldest group aged ≥ 75 years. This differed from the U.S. population in whom the proportion of diabetes-related complications increased in people aged 45–64 years but decreased in older people aged ≥ 65 years [39]. The results in our study might reflect a combined effect of increased proportion of the oldest people aged ≥ 75 years living with diabetes in Hong Kong and a delay in onset of complications. This observation has important implications for utilization of health services given the large and continually increasing number of older people with diabetes in Hong Kong and the profound economic burden of diabetes and its complications. For LEA, middle-aged adults (45–64 years) accounted for a greater proportion of LEA events in 2016 compared to 2001, although the event rate has declined. The reasons are unclear but may reflect a number of factors including patients’ preference for earlier amputations to prevent more serious functional loss, improved health resources with earlier referral, and changes in doctors’ attitude of whether to amputate. Further studies are needed to clarify this.

### Strengths and limitations

The major strengths of this study include the territory-wide coverage with minimal selection bias, and a long surveillance period. There are several limitations of this study. Firstly, our findings were restricted to people attending hospitals and clinics in the public sector. However, given the heavily subsidized public healthcare system, the HA provides over 90% of the inpatient services in Hong Kong [27]. The exclusion of the very small proportion of people who presented to the private sector should have little effect on the overall trend estimates and is unlikely to have seriously biased our conclusions. Secondly, due to the lack of information on people’s medical records before 2000, we were not able to distinguish between the first incident and recurrent events. As a result, we were unable to ascertain whether the declines in event rates of diabetes-related complications were due to decreasing number of people with new-onset events or due to people having fewer recurrent events. Thirdly, as information on secondary diagnosis codes was not available, major events that have occurred but listed as secondary diagnoses were not included in our study leading to potential underestimation of events. Fourthly, a trend in earlier detection of diabetes through diagnostic improvement and screening approaches might have produced a lead time bias. More people with screen-detected diabetes contributed to the denominator in recent compared to earlier years, and a temporal decline in event rate of complications could be detected even if the event has occurred at the same time point along the disease trajectory without being delayed. However, exclusion of people with newly diagnosed diabetes in each year did not affect the trends in rates of diabetes-related complications. Fifthly, the HKDSD is derived from an administrative source with a potential limitation in validity. We could not rule out the possibility of including people who used glucose-lowering drugs for reasons other than diabetes such as prediabetes and polycystic ovarian syndrome, although the number is deemed small. Additionally, we were not able to differentiate between the types of diabetes in people with diabetes onset before 2002. In our sensitivity analysis, removal of people with type 1 diabetes of onset between 2002 and 2015 as identified using a validated algorithm did not affect the trend estimates [40]. Lastly, we did not include all diabetes-related complications in our study. Future studies are needed to explore the trends of other important diabetes-related complications such as end stage kidney disease and severe retinopathy.

## Conclusions

Using surveillance data, we reported significant declines in event rates of major diabetes-related complications among people with diabetes in Hong Kong, but not in young adults, highlighting the importance of improving quality of care to prevent complications in young people at risk. Although the event rates have fallen, the absolute number of events has not decreased, and there were progressive increases in the contribution to most diabetes-related complications from the oldest age group. Given the increasing life expectancy of people with diabetes and the vulnerability of older individuals to cardiovascular diseases, healthcare resources should be appropriately prioritized to support management of these complications to reduce disability and maximise quality of life.

## Supplementary information

**Additional file 1: Figure S1.** Number of men and women with diabetes in the middle of the year by age in Hong Kong between 2001 and 2016. **Figure S2.** Number of events of diabetes-related complications in men and women with diabetes in Hong Kong between 2001 and 2016. **Figure S3.** Age-standardized event rates of diabetes-related complications in sensitivity analysis excluding people who were newly included in the HKDSD in each study year. **Figure S4.** Proportion of coronary heart disease events by age group in men and women with diabetes in Hong Kong between 2001 and 2016. **Figure S5.** Proportion of stroke events by age group in men and women with diabetes in Hong Kong between 2001 and 2016. **Figure S6.** Proportion of heart failure events by age group in men and women with diabetes in Hong Kong between 2001 and 2016. **Figure S7.** Proportion of hyperglycaemic crisis events by age group in men and women with diabetes in Hong Kong between 2001 and 2016. **Figure S8.** Proportion of amputation events by age group in men and women with diabetes in Hong Kong between 2001 and 2016. **Table S1.** Characteristics of people in the HKDSD between 2001 and 2016. **Table S2.** Age-standardized event rates (per 10,000) of diabetes-related complications in men with diabetes by age in Hong Kong between 2001 and 2016. **Table S3.** Age-standardized event rates (per 10,000) of diabetes-related complications in women with diabetes by age in Hong Kong between 2001 and 2016. **Table S4.** Joinpoint analysis of trends in age-standardized event rates of minor and major LEA in men and women with diabetes in Hong Kong between 2001 and 2016. **Table S5.** Age-standardized prevalence (%) of statin use in people with diabetes by sex and age in Hong Kong between 2001 and 2016. **Table S6.** Age-standardized prevalence (%) of DPP-4 inhibitors, GLP-1 receptor agonists, and SGLT-2 inhibitors use in people with diabetes by sex in Hong Kong between 2001 and 2016.

## Data Availability

The datasets used during the current study are available from the corresponding author on reasonable request.

## Abbreviations

AAPC: Average annual percent change
APC: Annual percent change
CHD: Coronary heart disease
DPP-4: Dipeptidyl-peptidase-4
EMR: Electronic medical record
GLP-1: Glucagon-like peptide-1
HA: Hospital Authority
HbA1c: Glycated haemoglobin
HKDSD: Hong Kong Diabetes Surveillance Database
ICD-9: International Classification of Diseases Ninth Revision
LDL: Low-density lipoprotein
LEA: Lower-extremity amputation
RAMP: Risk Assessment and Management Program
SGLT-2: Sodium-glucose co-transporter-2
U.S.: United States
