# Supplementary material for: Trends in diabetes-related complications in Hong Kong, 2001–2016: a retrospective cohort study
Source: Cardiovasc Diabetol. 2020 May 12;19:60. doi: 10.1186/s12933-020-01039-y (PMC7218631; doi:10.1186/s12933-020-01039-y)
Supplement: Supplementary file 1 — Additional file 1: Figure S1. Number of men and women with diabetes in the middle of the year by age in Hong Kong between 2001 and 2016. Figure S2. Number of events of diabetes-related complications in men and women with diabetes in Hong Kong between 2001 and 2016. Figure S3. Age-standardized event rates of diabetes-related complications in sensitivity analysis excluding people who were newly included in the HKDSD in each study year. Figure S4. Proportion of coronary heart disease events by age group in men and women with diabetes in Hong Kong between 2001 and 2016. Figure S5. Proportion of stroke events by age group in men and women with diabetes in Hong Kong between 2001 and 2016. Figure S6. Proportion of heart failure events by age group in men and women with diabetes in Hong Kong between 2001 and 2016. Figure S7. Proportion of hyperglycaemic crisis events by age group in men and women with diabetes in Hong Kong between 2001 and 2016. Figure S8. Proportion of amputation events by age group in men and women with diabetes in Hong Kong between 2001 and 2016. Table S1. Characteristics of people in the HKDSD between 2001 and 2016. Table S2. Age-standardized event rates (per 10,000) of diabetes-related complications in men with diabetes by age in Hong Kong between 2001 and 2016. Table S3. Age-standardized event rates (per 10,000) of diabetes-related complications in women with diabetes by age in Hong Kong between 2001 and 2016. Table S4. Joinpoint analysis of trends in age-standardized event rates of minor and major LEA in men and women with diabetes in Hong Kong between 2001 and 2016. Table S5. Age-standardized prevalence (%) of statin use in people with diabetes by sex and age in Hong Kong between 2001 and 2016. Table S6. Age-standardized prevalence (%) of DPP-4 inhibitors, GLP-1 receptor agonists, and SGLT-2 inhibitors use in people with diabetes by sex in Hong Kong between 2001 and 2016. [file 12933_2020_1039_MOESM1_ESM.docx]

**Figure S1. Number of men and women with diabetes in the middle of the year by age in Hong Kong between 2001 and 2016**


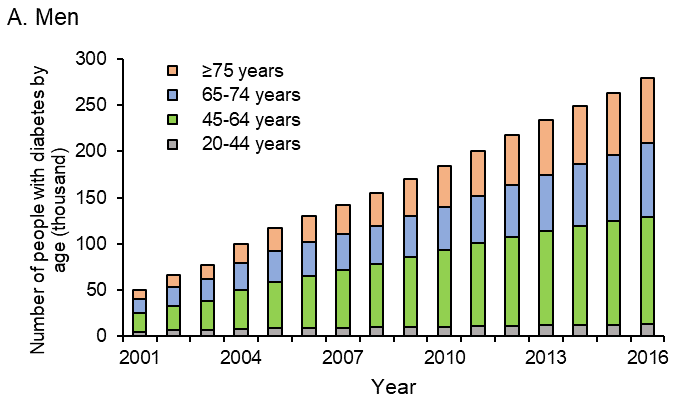

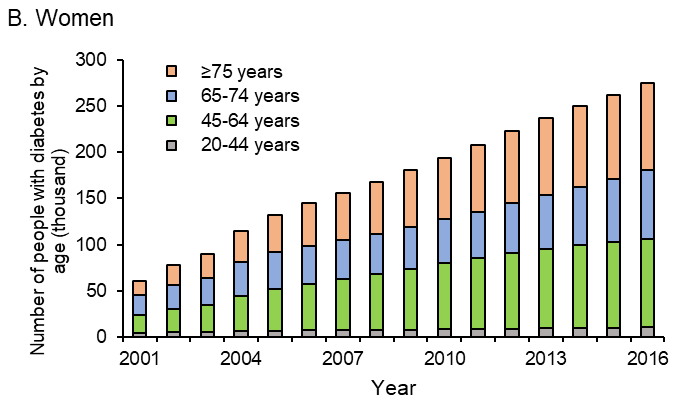


**Figure S2. Number of events of diabetes-related complications in men and women with diabetes in Hong Kong between 2001 and 2016**


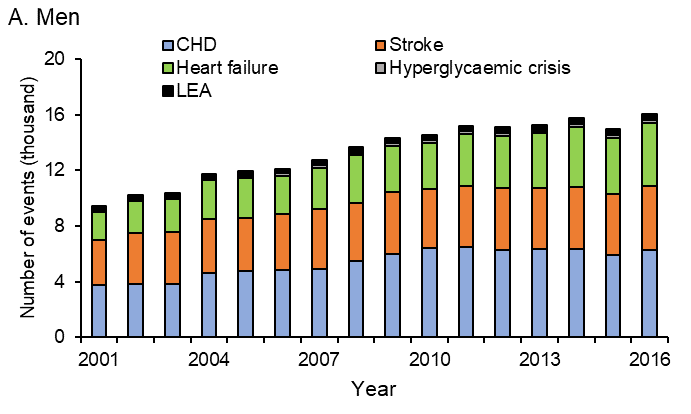

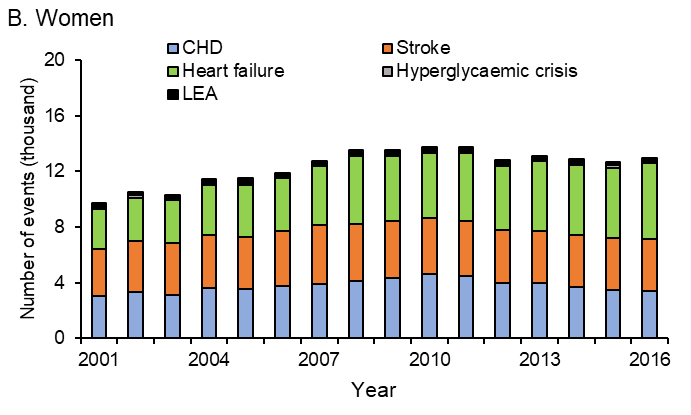


CHD, coronary heart disease; LEA, lower-extremity amputation

**Figure S3.** **Age-standardized event rates of diabetes-related complications in sensitivity analysis excluding people who were newly included in the HKDSD in each study year**

CHD, coronary heart disease; LEA, lower-extremity amputation. Dots are observed event rates. Solid lines are modeled event rates from the Joinpoint regression analysis.

**Figure S4. Proportion of coronary heart disease events by age group in men and women with diabetes in Hong Kong between 2001 and 2016**


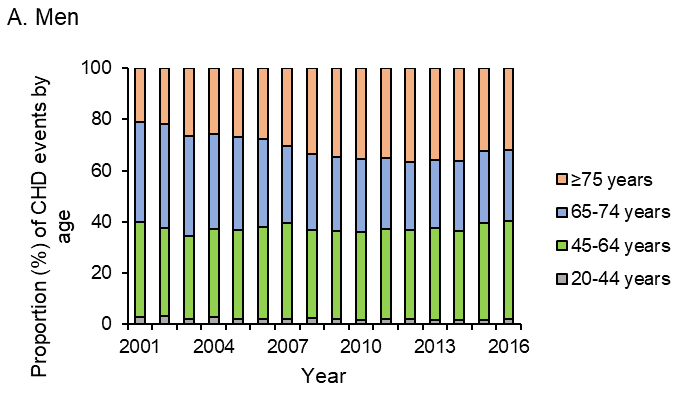

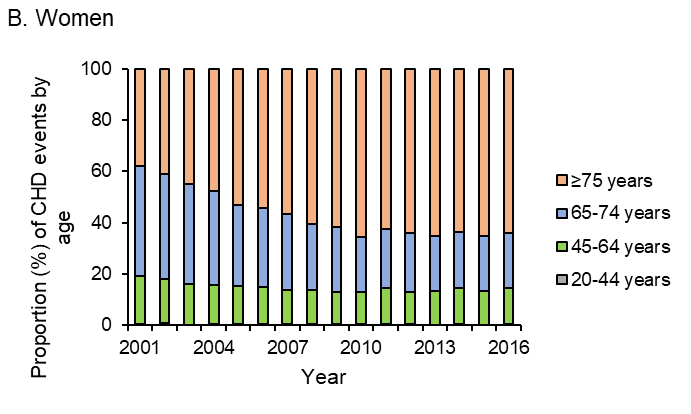


**Figure S5. Proportion of stroke events by age group in men and women with diabetes in Hong Kong between 2001 and 2016**


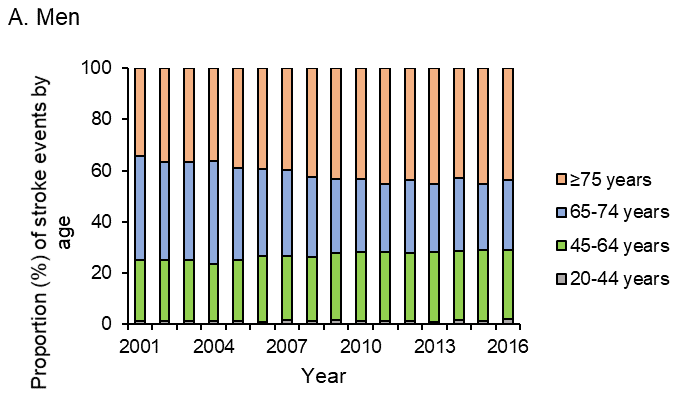

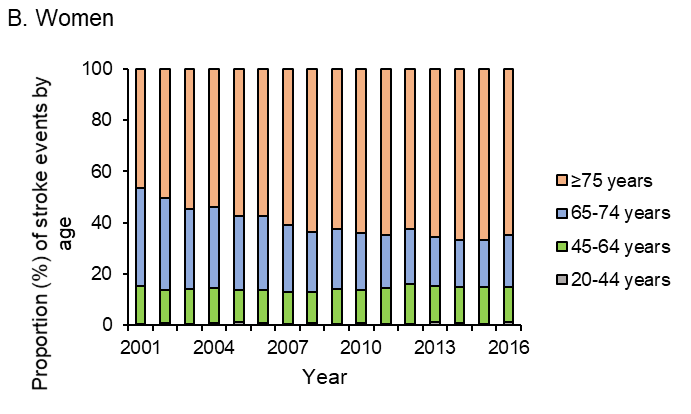


**Figure S6. Proportion of heart failure events by age group in men and women with diabetes in Hong Kong between 2001 and 2016**


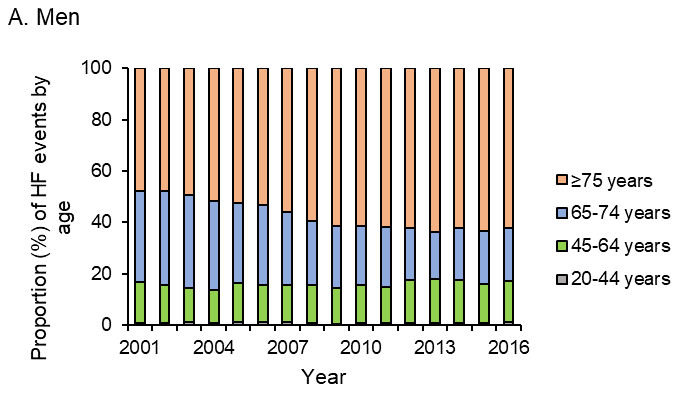

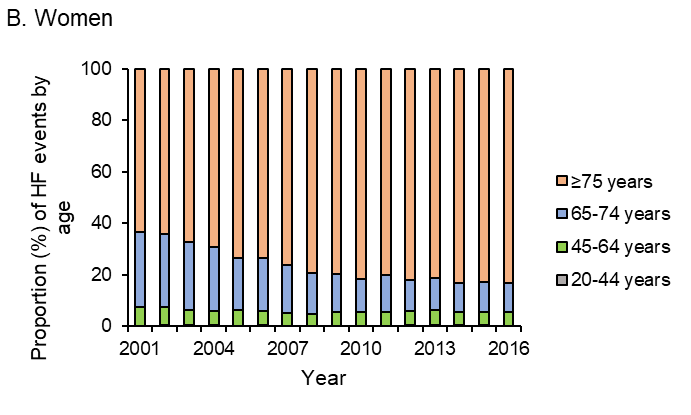


**Figure S7. Proportion of hyperglycaemic crisis events by age group in men and women with diabetes in Hong Kong between 2001 and 2016**


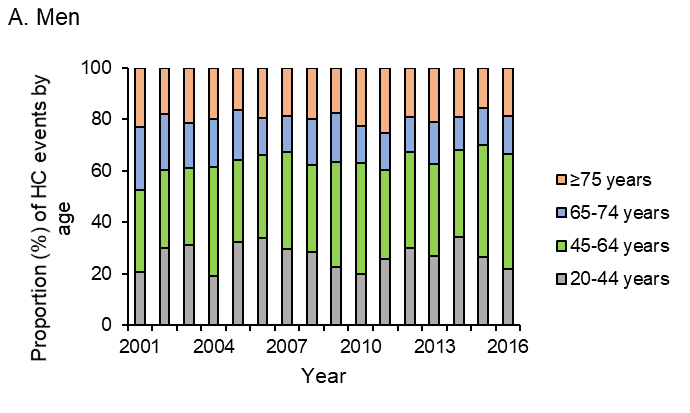

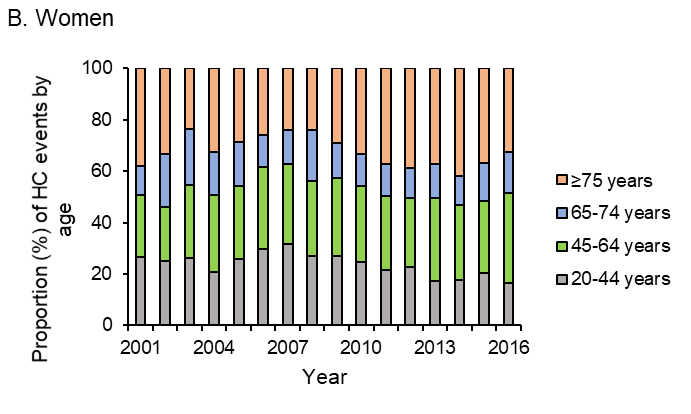


**Figure S8. Proportion of amputation events by age group in men and women with diabetes in Hong Kong between 2001 and 2016**


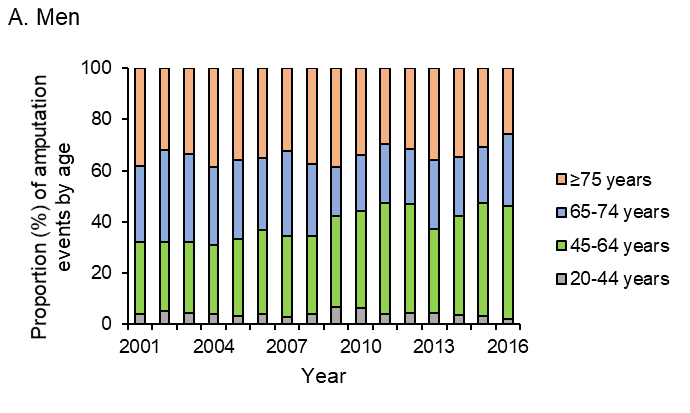

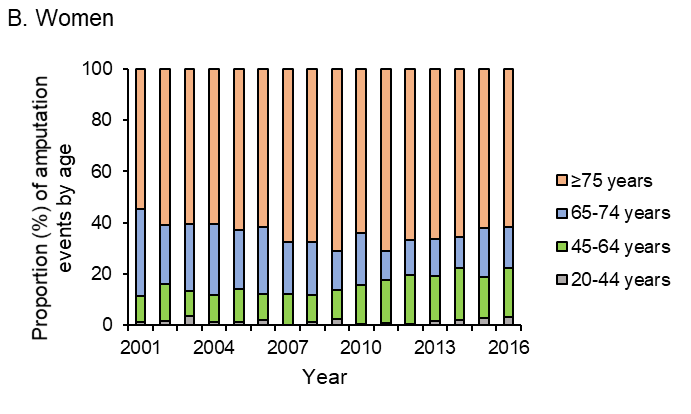


**Table S1. Characteristics of people in the HKDSD between 2001 and 2016**

|  | 2001 | 2002 | 2003 | 2004 | 2005 | 2006 | 2007 | 2008 | 2009 | 2010 | 2011 | 2012 | 2013 | 2014 | 2015 | 2016 |
| --- | --- | --- | --- | --- | --- | --- | --- | --- | --- | --- | --- | --- | --- | --- | --- | --- |
| Men |  |  |  |  |  |  |  |  |  |  |  |  |  |  |  |  |
| Number in the middle of the year | 52,625 | 68,865 | 80,287 | 102,496 | 119,608 | 135,859 | 148,542 | 161,769 | 176,349 | 191,431 | 207,775 | 224,461 | 240,527 | 255,876 | 270,611 | 285,988 |
| Mean age at the study year (years) (SD) | 63.2 (12.9) | 63.3 (12.9) | 63.4 (12.8) | 63.5 (12.6) | 63.7 (12.6) | 63.9 (12.6) | 64.0 (12.6) | 64.2 (12.6) | 64.3 (12.6) | 64.5 (12.6) | 64.8 (12.6) | 65.2 (12.5) | 65.2 (12.5) | 65.4 (12.5) | 65.7 (12.4) | 65.9 (12.4) |
| Mean fasting glucose (mol/L) (SD) | 8.0 (3.1) | 7.8 (3.0) | 7.9 (2.9) | 7.8 (2.9) | 7.6 (2.8) | 7.7 (2.8) | 7.6 (2.7) | 7.6 (2.7) | 7.5 (2.7) | 7.4 (2.5) | 7.3 (2.5) | 7.3 (2.4) | 7.4 (2.4) | 7.4 (2.5) | 7.5 (2.5) | 7.5 (2.5) |
| Mean HbA1c (mmol/mol) (SD) | 61.5 (7.8) | 60.9 (7.7) | 61.3 (7.8) | 61.4 (7.8) | 61.5 (7.8) | 61.3 (7.8) | 61.3 (7.8) | 59.3 (7.6) | 58.9 (7.5) | 57.6 (7.4) | 57.8 (7.4) | 57.4 (7.4) | 57.1 (7.4) | 56.1 (7.3) | 56.3 (7.3) | 56.9 (7.4) |
| Mean LDL-cholesterol (mmol/L) (SD) | 3.2 (1.0) | 3.2 (1.0) | 3.0 (1.0) | 3.0 (1.0) | 2.9 (0.9) | 2.9 (0.9) | 2.9 (0.9) | 2.9 (0.9) | 2.8 (0.9) | 2.8 (0.9) | 2.7 (0.9) | 2.6 (0.8) | 2.6 (0.8) | 2.5 (0.8) | 2.4 (0.8) | 2.3 (0.8) |
|  |  |  |  |  |  |  |  |  |  |  |  |  |  |  |  |  |
| Women |  |  |  |  |  |  |  |  |  |  |  |  |  |  |  |  |
| Number in the middle of the year | 64,697 | 82,269 | 93,390 | 118,272 | 135,804 | 152,888 | 164,561 | 176,288 | 189,460 | 202,925 | 216,822 | 231,975 | 246,055 | 259,440 | 271,904 | 284,941 |
| Mean age at the study year (years) (SD) | 66.1 (13.0) | 66.4 (13.1) | 66.6 (13.1) | 66.6 (12.9) | 66.9 (12.9) | 67.0 (13.0) | 67.1 (13.0) | 67.3 (13.1) | 67.4 (13.2) | 67.6 (13.2) | 67.7 (13.2) | 67.9 (13.2) | 68.0 (13.2) | 68.2 (13.2) | 68.4 (13.3) | 68.6 (13.3) |
| Mean fasting glucose (mol/L) (SD) | 8.0 (3.0) | 7.9 (2.9) | 7.9 (2.9) | 7.8 (2.9) | 7.7 (2.8) | 7.7 (2.8) | 7.6 (2.8) | 7.6 (2.7) | 7.6 (2.6) | 7.4 (2.6) | 7.3 (2.5) | 7.2 (2.4) | 7.3 (2.4) | 7.4 (2.4) | 7.4 (2.4) | 7.4 (2.4) |
| Mean HbA1c (mmol/mol) (SD) | 60.5 (7.7) | 60.0 (7.6) | 60.4 (7.7) | 60.7 (7.7) | 61.1 (7.7) | 60.7 (7.7) | 60.8 (7.7) | 58.7 (7.5) | 58.3 (7.5) | 57.0 (7.4) | 57.5 (7.4) | 57.1 (7.4) | 56.7 (7.3) | 55.7 (7.2) | 55.9 (7.3) | 56.3 (7.3) |
| Mean LDL-cholesterol (mmol/L) (SD) | 3.3 (1.0) | 3.3 (1.0) | 3.2 (1.0) | 3.2 (1.0) | 3.0 (1.0) | 3.1 (1.0) | 3.1 (1.0) | 3.0 (1.0) | 3.0 (0.9) | 3.0 (0.9) | 2.8 (0.9) | 2.7 (0.9) | 2.7 (0.9) | 2.6 (0.9) | 2.5 (0.8) | 2.4 (0.8) |

**SD, standard deviation; HbA1c, glycated haemoglobin; LDL, low-density lipoprotein**

**Table S2. Age-standardized event rates (per 10,000) of diabetes-related complications in men with diabetes by age in Hong Kong between 2001 and 2016**

|  | 2001 | 2002 | 2003 | 2004 | 2005 | 2006 | 2007 | 2008 | 2009 | 2010 | 2011 | 2012 | 2013 | 2014 | 2015 | 2016 |
| --- | --- | --- | --- | --- | --- | --- | --- | --- | --- | --- | --- | --- | --- | --- | --- | --- |
| CHD |  |  |  |  |  |  |  |  |  |  |  |  |  |  |  |  |
| 20-44 years | 155.9 | 126.6 | 71.9 | 85.5 | 54.0 | 60.1 | 64.5 | 79.4 | 56.9 | 73.6 | 91.6 | 62.0 | 58.8 | 56.8 | 38.6 | 55.1 |
| 45-64 years | 651.9 | 462.2 | 377.3 | 370.6 | 318.1 | 296.8 | 277.9 | 256.3 | 257.7 | 255.6 | 245.1 | 221.4 | 218.6 | 203.5 | 194.9 | 199.8 |
| 65-74 years | 914.3 | 746.0 | 614.1 | 568.0 | 496.9 | 449.6 | 377.6 | 383.9 | 387.0 | 381.4 | 347.5 | 296.4 | 278.6 | 256.6 | 227.6 | 217.1 |
| ≥75 years | 813.3 | 630.9 | 614.9 | 590.8 | 517.4 | 469.2 | 470.6 | 513.8 | 524.0 | 533.4 | 471.6 | 435.8 | 394.5 | 367.1 | 289.8 | 281.1 |
| Stroke |  |  |  |  |  |  |  |  |  |  |  |  |  |  |  |  |
| 20-44 years | 56.2 | 59.9 | 34.6 | 42.2 | 36.7 | 29.6 | 46.0 | 30.8 | 45.1 | 40.1 | 26.7 | 33.4 | 30.0 | 29.4 | 27.5 | 50.3 |
| 45-64 years | 338.2 | 300.7 | 257.0 | 191.1 | 165.7 | 169.5 | 161.2 | 142.9 | 145.3 | 128.1 | 122.6 | 113.6 | 105.7 | 102.6 | 103.0 | 99.7 |
| 65-74 years | 788.5 | 651.2 | 585.7 | 508.3 | 386.9 | 362.7 | 354.1 | 310.3 | 287.5 | 249.8 | 222.2 | 222.1 | 188.8 | 187.3 | 153.0 | 157.5 |
| ≥75 years | 1224.8 | 1070.8 | 937.4 | 757.3 | 649.3 | 614.1 | 556.9 | 519.1 | 504.5 | 434.3 | 420.0 | 371.9 | 341.0 | 306.4 | 295.9 | 289.0 |
| Heart failure |  |  |  |  |  |  |  |  |  |  |  |  |  |  |  |  |
| 20-44 years | 41.6 | 21.4 | 19.2 | 14.8 | 24.0 | 24.3 | 51.4 | 30.8 | 23.8 | 19.6 | 20.9 | 24.1 | 18.2 | 18.3 | 31.1 | 30.8 |
| 45-64 years | 147.5 | 112.0 | 91.8 | 77.8 | 81.9 | 66.3 | 62.7 | 70.3 | 57.6 | 53.3 | 53.9 | 58.2 | 58.9 | 61.5 | 51.1 | 56.6 |
| 65-74 years | 431.9 | 369.2 | 339.2 | 302.0 | 241.0 | 220.9 | 200.6 | 197.0 | 165.9 | 149.0 | 159.7 | 128.2 | 115.8 | 126.2 | 113.3 | 116.4 |
| ≥75 years | 1259.2 | 986.9 | 842.2 | 786.1 | 691.6 | 603.7 | 584.5 | 663.9 | 563.2 | 517.4 | 528.8 | 470.4 | 455.7 | 453.0 | 402.3 | 413.4 |
| Hyperglycaemic crisis |  |  |  |  |  |  |  |  |  |  |  |  |  |  |  |  |
| 20-44 years | 151.7 | 144.1 | 82.9 | 61.5 | 103.8 | 96.9 | 102.6 | 75.4 | 67.2 | 52.6 | 65.6 | 83.0 | 74.3 | 85.7 | 67.2 | 66.0 |
| 45-64 years | 25.4 | 22.5 | 11.9 | 13.3 | 9.7 | 8.6 | 10.6 | 7.9 | 10.4 | 10.3 | 8.1 | 8.6 | 7.7 | 8.5 | 8.5 | 10.1 |
| 65-74 years | 23.2 | 19.4 | 6.9 | 8.0 | 8.4 | 6.1 | 5.8 | 5.3 | 8.1 | 5.6 | 5.0 | 4.6 | 5.0 | 3.9 | 3.7 | 4.0 |
| ≥75 years | 42.3 | 31.0 | 19.1 | 15.0 | 10.6 | 13.6 | 10.4 | 7.6 | 8.6 | 9.7 | 9.6 | 7.6 | 6.1 | 5.8 | 4.5 | 5.5 |
| LEA |  |  |  |  |  |  |  |  |  |  |  |  |  |  |  |  |
| 20-44 years | 14.1 | 13.4 | 13.9 | 17.6 | 9.8 | 8.5 | 9.0 | 16.9 | 18.7 | 16.4 | 10.1 | 10.5 | 8.6 | 9.0 | 7.3 | 3.8 |
| 45-64 years | 37.5 | 26.1 | 25.1 | 23.2 | 21.1 | 18.3 | 18.4 | 19.0 | 18.1 | 19.4 | 19.5 | 21.4 | 12.3 | 18.1 | 18.6 | 18.4 |
| 65-74 years | 48.0 | 47.4 | 41.2 | 37.2 | 31.9 | 24.9 | 31.4 | 29.5 | 15.8 | 19.2 | 17.4 | 18.3 | 16.9 | 15.7 | 13.6 | 16.4 |
| ≥75 years | 123.0 | 75.6 | 70.7 | 81.3 | 57.6 | 41.2 | 41.4 | 49.3 | 41.5 | 34.9 | 24.5 | 29.8 | 23.9 | 26.2 | 21.0 | 16.6 |

CHD, coronary heart disease; LEA, lower-extremity amputation

**Table S3. Age-standardized event rates (per 10,000) of diabetes-related complications in women with diabetes by age in Hong Kong between 2001 and 2016**

|  | 2001 | 2002 | 2003 | 2004 | 2005 | 2006 | 2007 | 2008 | 2009 | 2010 | 2011 | 2012 | 2013 | 2014 | 2015 | 2016 |
| --- | --- | --- | --- | --- | --- | --- | --- | --- | --- | --- | --- | --- | --- | --- | --- | --- |
| CHD |  |  |  |  |  |  |  |  |  |  |  |  |  |  |  |  |
| 20-44 years | 18.6 | 25.7 | 6.7 | 13.4 | 13.2 | 12.7 | 9.1 | 27.3 | 19.0 | 14.9 | 23.6 | 10.6 | 8.7 | 11.3 | 4.8 | 12.5 |
| 45-64 years | 240.7 | 197.3 | 150.1 | 127.5 | 101.5 | 95.3 | 82.5 | 75.7 | 71.7 | 71.0 | 69.0 | 55.2 | 55.5 | 49.0 | 42.8 | 43.0 |
| 65-74 years | 623.5 | 508.2 | 395.4 | 342.5 | 268.9 | 253.0 | 248.0 | 228.2 | 219.0 | 193.8 | 186.3 | 161.6 | 140.3 | 124.6 | 106.0 | 94.0 |
| ≥75 years | 727.3 | 642.0 | 552.8 | 522.4 | 479.6 | 455.4 | 446.9 | 457.9 | 452.4 | 462.1 | 394.8 | 333.1 | 310.4 | 267.9 | 243.9 | 223.2 |
| Stroke |  |  |  |  |  |  |  |  |  |  |  |  |  |  |  |  |
| 20-44 years | 41.5 | 55.7 | 34.7 | 28.2 | 44.1 | 24.3 | 23.0 | 27.8 | 20.5 | 26.4 | 16.1 | 14.4 | 25.8 | 23.0 | 18.9 | 28.2 |
| 45-64 years | 216.1 | 170.4 | 160.4 | 123.8 | 97.6 | 95.8 | 86.1 | 75.3 | 77.1 | 65.6 | 66.3 | 63.3 | 54.2 | 54.5 | 56.6 | 52.3 |
| 65-74 years | 590.2 | 463.7 | 380.8 | 304.4 | 255.1 | 260.1 | 244.3 | 202.5 | 194.0 | 177.9 | 159.9 | 138.1 | 116.7 | 102.6 | 97.8 | 100.3 |
| ≥75 years | 1044.8 | 909.2 | 821.2 | 643.8 | 565.7 | 525.2 | 529.6 | 493.4 | 435.7 | 399.5 | 368.3 | 304.7 | 293.8 | 277.7 | 268.8 | 249.7 |
| Heart failure |  |  |  |  |  |  |  |  |  |  |  |  |  |  |  |  |
| 20-44 years | 9.2 | 9.8 | 29.5 | 9.8 | 1.1 | 18.1 | 6.4 | 17.2 | 9.2 | 8.3 | 9.2 | 11.1 | 20.3 | 23.4 | 13.5 | 13.9 |
| 45-64 years | 87.4 | 74.4 | 57.9 | 46.7 | 47.1 | 38.6 | 34.2 | 36.5 | 33.5 | 29.1 | 27.6 | 27.3 | 30.8 | 23.5 | 23.9 | 25.6 |
| 65-74 years | 371.0 | 297.2 | 258.5 | 217.8 | 167.9 | 167.3 | 162.3 | 159.2 | 134.0 | 118.0 | 129.6 | 96.6 | 99.4 | 82.0 | 81.8 | 79.1 |
| ≥75 years | 1273.3 | 1056.5 | 918.9 | 844.6 | 776.0 | 673.3 | 710.0 | 760.0 | 662.7 | 615.0 | 575.5 | 504.5 | 497.6 | 476.1 | 440.9 | 454.3 |
| Hyperglycaemic crisis |  |  |  |  |  |  |  |  |  |  |  |  |  |  |  |  |
| 20-44 years | 225.1 | 173.4 | 104.8 | 95.7 | 128.5 | 83.2 | 119.5 | 86.5 | 81.0 | 85.4 | 57.4 | 75.9 | 55.0 | 48.8 | 59.0 | 36.0 |
| 45-64 years | 22.6 | 16.8 | 12.6 | 12.2 | 13.5 | 10.2 | 9.4 | 7.9 | 8.5 | 8.0 | 7.0 | 8.2 | 8.1 | 6.2 | 7.0 | 10.0 |
| 65-74 years | 8.5 | 15.4 | 9.6 | 6.5 | 6.2 | 3.4 | 3.9 | 6.3 | 4.9 | 4.3 | 3.8 | 3.6 | 3.8 | 2.8 | 4.2 | 4.0 |
| ≥75 years | 48.7 | 31.4 | 12.5 | 17.0 | 13.3 | 8.7 | 6.9 | 7.0 | 7.1 | 8.5 | 7.8 | 9.7 | 7.6 | 7.5 | 7.5 | 6.2 |
| LEA |  |  |  |  |  |  |  |  |  |  |  |  |  |  |  |  |
| 20-44 years | 9.4 | 5.8 | 9.4 | 6.0 | 6.2 | 3.5 | 0.0 | 3.4 | 8.1 | 1.5 | 2.5 | 0.5 | 2.3 | 4.1 | 4.0 | 4.9 |
| 45-64 years | 11.9 | 13.1 | 7.6 | 8.1 | 7.6 | 4.6 | 5.6 | 4.9 | 5.6 | 6.4 | 5.7 | 6.0 | 4.8 | 5.0 | 3.5 | 5.2 |
| 65-74 years | 39.2 | 20.1 | 20.1 | 19.3 | 16.2 | 13.6 | 9.4 | 13.3 | 8.5 | 13.3 | 5.5 | 5.7 | 5.4 | 5.2 | 6.0 | 5.1 |
| ≥75 years | 102.2 | 80.8 | 66.0 | 55.0 | 52.9 | 37.8 | 37.2 | 37.5 | 35.9 | 32.0 | 30.0 | 21.3 | 17.6 | 19.3 | 14.1 | 14.4 |

CHD, coronary heart disease; LEA, lower-extremity amputation

**Table S4. Joinpoint analysis of trends in age-standardized event rates of minor and major LEA in men and women with diabetes in Hong Kong between 2001 and 2016**

|  | Event rates (per 10,000) | | | Time period 1 | | Time period 2 | | Time period 3 | |
| --- | --- | --- | --- | --- | --- | --- | --- | --- | --- |
|  | 2001 | 2016 | AAPC (95% CI) | Year | APC (95% CI) | Year | APC (95% CI) | Year | APC (95% CI) |
| Minor LEA |  |  |  |  |  |  |  |  |  |
| Men | 14.0 | 7.2 | -3.8 (-5.7, -1.9)* | 2001-2016 | -3.8 (-5.7, -1.9)* |  |  |  |  |
| Women | 7.9 | 3.2 | -6.3 (-10.6, -1.8)* | 2001-2016 | -6.3 (-10.6, -1.8)* |  |  |  |  |
|  |  |  |  |  |  |  |  |  |  |
| Major LEA |  |  |  |  |  |  |  |  |  |
| Men | 19.5 | 4.3 | -8.0 (-9.6, -6.5)* | 2001-2016 | -8.0 (-9.6, -6.5)* |  |  |  |  |
| Women | 11.6 | 2.4 | -10.4 (-13.1, -7.6)* | 2001-2006 | -16.4 (-22.1, -10.2)* | 2006-2016 | -7.2 (-10.6, -3.6)* |  |  |

LEA, lower-extremity amputation; AAPC: average annual percent change; APC: annual percent change. **P* <0.05

**Table S5. Age-standardized prevalence (%) of statin use in people with diabetes by sex and age in Hong Kong between 2001 and 2016**

|  | 2001 | 2002 | 2003 | 2004 | 2005 | 2006 | 2007 | 2008 | 2009 | 2010 | 2011 | 2012 | 2013 | 2014 | 2015 | 2016 |
| --- | --- | --- | --- | --- | --- | --- | --- | --- | --- | --- | --- | --- | --- | --- | --- | --- |
| Men |  |  |  |  |  |  |  |  |  |  |  |  |  |  |  |  |
| All ages | 11.0 | 11.1 | 10.9 | 11.3 | 11.5 | 10.2 | 10.5 | 11.7 | 15.8 | 22.2 | 27.6 | 32.5 | 36.3 | 39.7 | 42.4 | 44.5 |
| 20-44 years | 6.6 | 6.5 | 6.5 | 7.8 | 7.4 | 5.8 | 5.3 | 5.8 | 9.0 | 12.9 | 16.0 | 19.4 | 21.4 | 23.6 | 26.1 | 27.6 |
| 45-64 years | 15.2 | 15.1 | 14.5 | 13.9 | 14.2 | 12.5 | 13.1 | 14.6 | 19.4 | 28.1 | 35.3 | 41.4 | 46.5 | 50.6 | 53.4 | 55.7 |
| 65-74 years | 16.4 | 17.0 | 17.0 | 16.9 | 18.0 | 17.5 | 18.9 | 20.7 | 25.8 | 34.4 | 42.2 | 48.7 | 54.0 | 58.6 | 61.8 | 64.3 |
| ≥75 years | 8.5 | 9.7 | 10.4 | 11.7 | 12.9 | 13.5 | 15.5 | 18.3 | 22.4 | 28.6 | 35.0 | 40.5 | 45.5 | 49.9 | 53.7 | 56.8 |
| Women |  |  |  |  |  |  |  |  |  |  |  |  |  |  |  |  |
| All ages | 10.4 | 10.2 | 10.1 | 9.8 | 9.5 | 7.4 | 7.6 | 8.5 | 12.4 | 19.5 | 25.4 | 30.3 | 34.4 | 37.0 | 39.0 | 40.2 |
| 20-44 years | 5.2 | 4.8 | 5.7 | 5.9 | 5.0 | 3.0 | 3.0 | 3.6 | 5.4 | 8.2 | 11.4 | 13.9 | 16.4 | 17.5 | 18.4 | 18.5 |
| 45-64 years | 14.2 | 14.0 | 12.7 | 11.9 | 11.7 | 9.0 | 8.8 | 9.7 | 15.1 | 25.6 | 33.7 | 40.4 | 45.9 | 49.4 | 51.9 | 53.6 |
| 65-74 years | 19.5 | 19.5 | 18.6 | 17.1 | 17.4 | 15.3 | 16.1 | 17.6 | 24.1 | 36.1 | 44.9 | 52.0 | 57.2 | 61.3 | 64.4 | 66.7 |
| ≥75 years | 9.4 | 10.1 | 10.7 | 11.6 | 12.9 | 12.8 | 14.6 | 17.3 | 21.9 | 29.7 | 36.5 | 42.5 | 47.8 | 52.4 | 56.2 | 59.0 |

**Table S6. Age-standardized prevalence (%) of DPP-4** **inhibitors, GLP-1 receptor agonists, and SGLT-2 inhibitors use in people with diabetes by sex in Hong Kong between 2001 and 2016**

|  | 2001 | 2002 | 2003 | 2004 | 2005 | 2006 | 2007 | 2008 | 2009 | 2010 | 2011 | 2012 | 2013 | 2014 | 2015 | 2016 |
| --- | --- | --- | --- | --- | --- | --- | --- | --- | --- | --- | --- | --- | --- | --- | --- | --- |
| Men |  |  |  |  |  |  |  |  |  |  |  |  |  |  |  |  |
| DPP-4 inhibitors |  |  |  |  |  |  | 0.01 | 0.05 | 0.30 | 0.62 | 1.72 | 2.98 | 3.89 | 5.36 | 6.71 | 7.77 |
| GLP-1 receptor agonists |  |  |  |  |  |  |  |  |  |  | 0.03 | 0.07 | 0.04 | 0.07 | 0.06 | 0.17 |
| SGLT-2 inhibitors |  |  |  |  |  |  |  |  |  |  |  |  |  |  | 0.11 | 0.80 |
|  |  |  |  |  |  |  |  |  |  |  |  |  |  |  |  |  |
| Women |  |  |  |  |  |  |  |  |  |  |  |  |  |  |  |  |
| DPP-4 inhibitors |  |  |  |  |  |  | 0.01 | 0.04 | 0.19 | 0.45 | 1.57 | 2.74 | 3.48 | 4.38 | 5.46 | 6.39 |
| GLP-1 receptor agonists |  |  |  |  |  |  |  |  |  |  | 0.04 | 0.07 | 0.09 | 0.06 | 0.09 | 0.13 |
| SGLT-2 inhibitors |  |  |  |  |  |  |  |  |  |  |  |  |  |  | 0.06 | 0.69 |

DPP-4, dipeptidyl-peptidase-4; GLP-1, glucagon-lie peptide-1; SGLT-2, sodium-glucose cotransporter-2.
